# Appendicitis in Young Children

**Published:** 1913-05-03

**Authors:** 


### MATERNITY AND MOTHERING.

**(Inquiries and Correspondence are invited.)**

### Appendicitis in Young Children.

This disease is fortunately uncommon, comparatively speaking, in early childhood and infancy, at least in this country. A German surgeon holds a contrary view, so far as his own district is concerned, for he says it is seven times commoner there than in adults. Certainly no such frequency prevails in Britain. This relative infrequency of appendicitis in young children is all the more thankworthy, because of two marked peculiarities of the disease at that age. First, it is remarkably difficult to diagnose, insidious in onset, and anomalous both in symptoms and signs. Secondly, it is even more dangerous than in older folks, and more often runs a fulminating course to swift and irretrievable disaster.

It has been said that the diagnosis of the disease is often extremely obscure and difficult. Even expert consulting surgeons may fail to make it in the early stages of the case, and general practitioners are even more liable to be misled because they come across so many cases which begin in an exactly similar way and clear up entirely under simple treatment. It is not suggested—indeed it would be most undesirable from every point of view—that parents and those who are responsible for the health or well-being of children should attempt to unravel the tangle of abdominal symptoms which so many children present, or that they should omit to secure competent advice in such cases. Yet it is important that people should recognise the potentialities of "indigestion," "chills on the liver," "bilious attacks," and similar causes of abdominal pains, vomiting, and so on, in young children. This is to run the risk of anxious parents mistaking every dietary indiscretion and its subsequent retribution, for appendicitis, which also is to be deprecated. But occasionally even qualified doctors make this mistake, and it is after all one on the right side.

To illustrate the insidious course and difficulty in diagnosis, it will suffice to recite two actual cases. A boy of three was subject to occasional attacks of vomiting, usually with constipation, especially after dietary errors to which he was very prone. He had had many of these attacks, to which his parents had ceased to attach any importance. One day after consuming a considerable quantity of jam he was sick and complained of abdominal pain. He was seen in the afternoon, by which time all pain had disappeared. Temperature and pulse-rate were normal, as was the abdomen in every part: the appendix region was especially investigated, with absolutely negative results. A fortnight later a similar attack began, and at first no doctor was called in. The next day he seemed ill, and advice was taken. It was then seen that the case was appendicitis and an operation was performed. Unfortunately perforation had already occurred, general peritonitis was present, and death ensued in two days.

Compare with this the following case. A boy of eight or nine complained at about 6 A.M. of sudden pain and vomiting: he had gone to bed perfectly well the night before. A doctor saw him at 7 A.M., diagnosed appendicitis, and operation took place at 8.30 A.M. An inflamed appendix, full of pus, was removed; it was just on the point of perforating. Rapid recovery followed.

There can be little doubt that in the first case the preliminary attack was due to a mild appendicitis, which in a few hours subsided so that no trace of its existence remained. Operation then would beyond doubt have saved the child's life: yet if every child which is sick and has a stomach-ache is to be operated on, the whole population will be appendix-less by ten years of age.

Cautley * distinguishes two types of appendicitis in nurslings. "It may be insidious in onset, with either diarrhœa or constipation, vomiting, slight fever, and wasting. This gradually passes on to higher temperature, increased vomiting, distension, and peritonitis." In the second type the symptoms are much more sudden and acute, as in older folk. Under eighteen months the disease, he says, is almost invariably fatal. Besides these types there are in somewhat older children undoubtedly many cases in which the attack is mild and unrecognised, often unrecognisable. Mild indigestion or a bilious attack is commonly diagnosed; and although some surgeons would argue that these are all cases of appendicitis in reality, there is no doubt that in many of them the attacks subside and are finally outgrown altogether.

What then are the cardinal symptoms of appendicitis in children in a straightforward kind of case of medium severity? Pain is usually the first to arise, and it is nearly always referred to the umbilicus, instead of to the right side of the abdomen as in adults. Vomiting is common at the onset, but generally soon subsides. Here again is a difficulty, for many children vomit on the very slightest of provocation. The temperature is usually raised, sometimes little, sometimes much; but a normal temperature is no sign of safety. The pulse-rate is practically always quickened, but not so much in proportion as in adults. Tenderness and rigidity are very common in the right iliac fossa; and a palpable swelling there, will of course arouse suspicion at once. Unluckily, the fulminating cases may present no such swelling at all. The child may lie with the right knee bent up in bed, and may cry or complain of pain when the leg is straightened out. This sign is also sometimes to be noticed in cases of acute rheumatism or hip disease. There are other signs, but many of them are as uncertain as those given above: the diagnosis rests on a consideration of all the information elicited, taken together.

* *Diseases of Children*, p. 341.

